# Plasma activated water triggers plant defence responses

**DOI:** 10.1038/s41598-020-76247-3

**Published:** 2020-11-05

**Authors:** Yuri Zambon, Nicoletta Contaldo, Romolo Laurita, Eva Várallyay, Alessandro Canel, Matteo Gherardi, Vittorio Colombo, Assunta Bertaccini

**Affiliations:** 1 Department of Agricultural and Food Sciences (DISTAL), Plant Pathology, Alma Mater Studiorum-University of Bologna, V. le Fanin, 40, 40127 Bologna, Italy; 2 Department of Industrial Engineering (DIN), Alma Mater Studiorum-University of Bologna, Via Terracini 24, 40131 Bologna, Italy; 3 Agricultural Biotechnology Institute, National Agricultural Research and Innovation Center, Szent-Györgyi A street 4, Gödöllő, 2100 Hungary; 4 Industrial Research Centre for Advanced Mechanics and Materials (C.I.R.I.-M.A.M.), Alma Mater Studiorum-University of Bologna, Via Terracini 24, 40131 Bologna, Italy

**Keywords:** Molecular biology, Plant sciences, Engineering

## Abstract

Nowadays, one of the main challenges is moving towards an eco-sustainable agriculture, able to preserve the food production through a reduced use of pesticides. Current global food sustenance by intensive agriculture is mainly based on economic crop monocultures and drastically reduces the biodiversity, increasing the yield losses due to the presence of biotic and abiotic stresses. A technology based on plasma activated water (PAW), characterized by the presence in liquid of reactive oxygen and nitrogen species, was tested to try to ensure yield stability also enhancing the plant resistance responses and to promote an eco-sustainable management of plant diseases. In PAW-treated micropropagated periwinkle shoots, periwinkle and grapevine plants, qRT-PCR and small RNAs high-throughput sequencing were used to analyse the differential expression of genes involved in the major plant defence pathways. The results indicate that PAW treatment enhances the plant defence responses and provide an encouraging framework for future applications in plant disease management programs.

## Introduction

Plant production directly or indirectly constitutes more than 97% of the human diet supplying food, fibres, energy and raw materials to the world growing population. The need of increasing food availability could be pursued through two complementary approaches: improving unit yields and decreasing production losses. Plant protection in general and crop disease management in particular, play a key role in the growing demand for food quality and quantity^1^. Recent studies have shown that diseases, pests and weeds reduce the global yield by 30–40%, mainly in the developing countries^2,3^. Intensive conventional agriculture has more than tripled the crop yield in the last century^4^; however the use of pesticides and mineral fertilisers has a negative impact on the environment through decreasing biodiversity, increasing pollution and eutrophication of water, and degrading soil quality^5^. Strategies that enhance plant immunity and promote growth of healthy plants have a great potential to reduce the impact of plant diseases, and therefore limit the use of pesticides. One of the strategies is represented by the use of immunity inducers, a class of compounds that can induce systemic acquired resistance in plants^6^. They can be either non-biological or biological active molecules, depending on their origin and include synthetic elicitors, plant immunity-inducing proteins, chitosan oligosaccharides and microorganisms^7^. In response to abiotic and biotic stresses, plants have an adaptive defence system producing reactive oxygen and nitrogen species (RONS) that, in low concentrations, act as signalling molecules^8^. Moreover, both biotic and abiotic stresses cause RONS bursts, which induce the production of secondary metabolites, acting very often as defence hormone precursors in plant cells. Plant endogenous RONS level is increased by the presence of pathogens, and this involves various processes such as the hypersensitivity response (HR), the accumulation of phytoalexins and the activation of defence genes^9^. These processes are controlled by key genes encoding transcription activators and repressors that regulate downstream stress-induced genes and pathways^10–12^.

To simultaneously enhance food security and reduce the environmental impact of agriculture, a novel approach focused on the use of plasma activated water (PAW) was investigated. The exposure of water to a cold atmospheric pressure plasma induces the production of both reactive oxygen and nitrogen species in the liquid phase, indeed PAW is characterized by the high concentration of long-lived RONS (e.g. hydrogen peroxide and nitrates) that participate in various signalling pathways in plants, which regulate metabolic processes, plant development, and stress responses^13^.

Indeed, in the recent years it has been proved that PAW treatment enhances plant growth^14^ and a few pioneering experiments focused on the mechanisms triggered by PAW treatment in inducing resistance in plants^15–17^. In this work PAW was used for the treatment of micropropagated periwinkle shoots, periwinkle and grapevine plants, to evaluate the presence of induced resistance by gene expression changes.

## Results and discussion

### Concentration of hydrogen peroxide and nitrates in PAW

Plasma treatment of sterile distilled water (SDW) induced the production of short-lived (having a lifetime ranging from a few µs to a few ms) and long lived species, such as nitrites (NO_2_^−^), nitrates (NO_3_^−^), hydrogen peroxide (H_2_O_2_) and a pH reduction^13^. The concentration of nitrites fell below the detection limit a few minutes after the plasma treatment as previously reported^16,18^, while hydrogen peroxide and nitrate concentrations increased and remained stable for 24 h even after freezing and defrosting. In Table 1 the concentrations of hydrogen peroxide, nitrate and pH of the PAW are reported.

**Table 1:** The pH values, hydrogen peroxide and nitrate concentrations in PAW.

	pH	H_2_O_2_ (mg × L^−1^)	NO_3_^−^ (mg × L^−1^)
SDW	5.5 ± 0.1	0	0
PAW	2.78 ± 0.47	13.5 ± 1.3	81.9 ± 3.4

### Phytoalexin pathway is induced in PAW treated micropropagated periwinkle shoots

**Figure 1:**
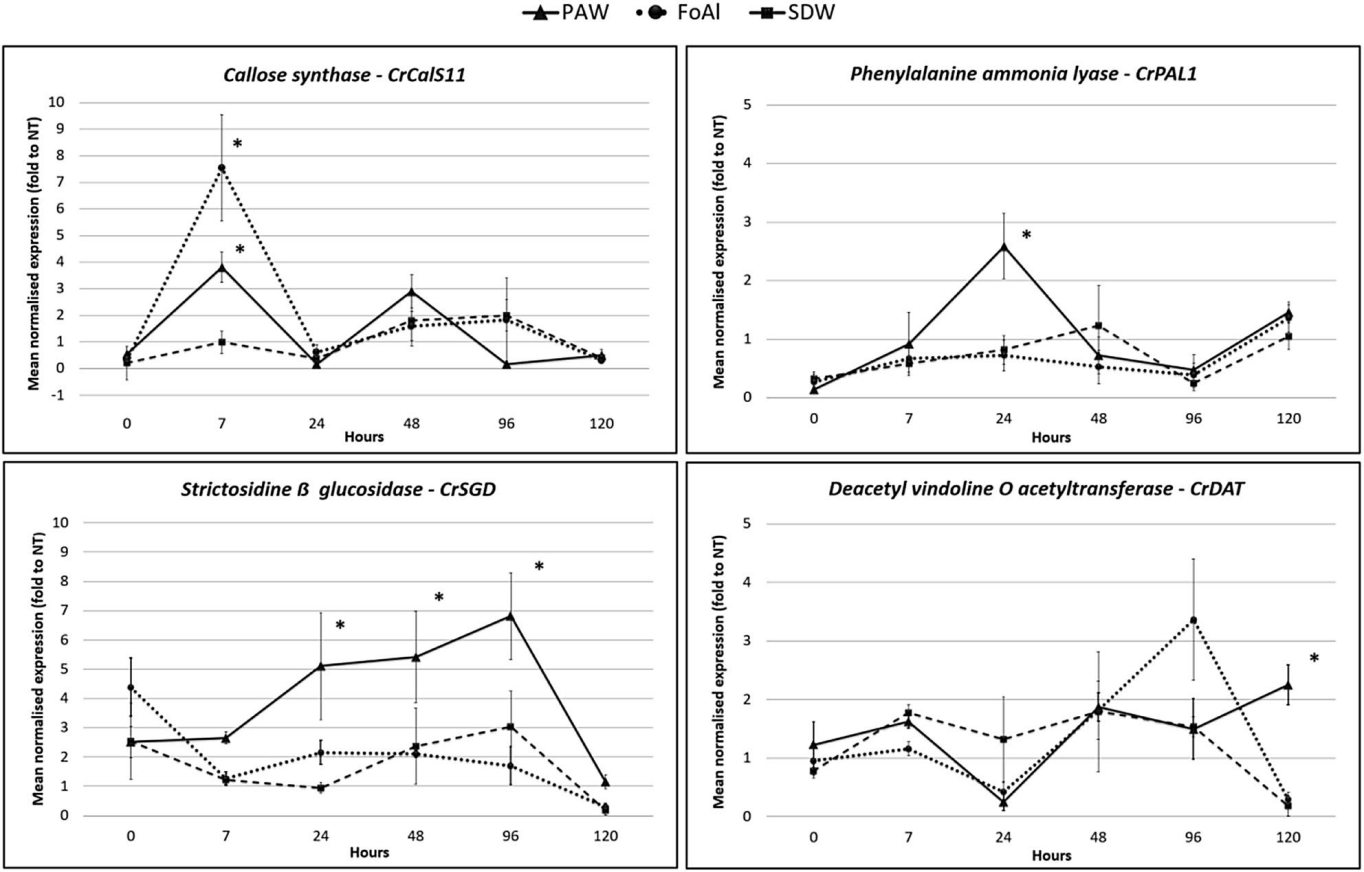
Gene expression changes in micropropagated periwinkle shoots after PAW treatment. Gene expression for callose synthase (*CrCalS11*), phenylalanine ammonia lyase (*CrPAL1*), strictosidine-β-glucosidase (*CrSGD*) and deacetyl vindoline-O-acetyltransferase (*CrDAT*) as determined by qRT-PCR after PAW, SDW and FoAl treatments. Their relative expression (ubiquitin was the internal control) to untreated shoots are shown at different time points after treatment. *Statistically significant *P* < 0.05.

Micropropagated periwinkle shoots treated with PAW and collected at different times after treatment were examined by quantitative PCR (qPCR) for gene expression changes. To minimize the possible negative effects of the treatments, all the plants considered were subjected to the submersion and neither hypoxic nor anoxic stress was observed. In PAW-treated micropropagated periwinkle shoots a statistically significant overexpression of the *CrCalS11*, *CrPAL1*, and *CrSGD* genes could be observed (Fig. 1). Moreover, a light overexpression of *CrDAT* gene was registered at a late time point (120 h post treatment, pt), while no significant different expression was observed for the *CrCHS* gene (Supplementary File 1). A significant increase in *CrCalS11* expression in shoots treated with both PAW and fosetyl aluminium (FoAl) was observed (3.8 and 7.5 fold, respectively) 7 h pt.

The callose deposition is induced by the activity of this gene and has been reported in several studies as a generic response to biotic and abiotic stresses ^19^. The overexpression of *CrCalS11* in response to PAW similarly to FoAl, indicates the induction of defence reactions and could be related to the presence of RONS and in particular H_2_O_2_ in the PAW solution^ 20–22^. Expression of *CrPAL1* in the PAW treated shoots increased by 2.6-fold at 24 h pt while the shoots treated with FoAl did not show any increase, very likely because of the absence of pathogen(s)^23^. *CrPAL1* overexpression as a result of shoots exposure to PAW is explained by the presence of H_2_O_2_ and NO_3_^−^ as stress response signals^24^. Nitric oxide (NO) and its donors activate the transcription of the PAL gene through a cGMP (guanosine-monophosphate cyclic)-dependent mechanism ^25^. *CrPAL1 *induction can also be the effect of the presence of H_2_O_2_, considered to be the main intermediary in the activation of the induced resistance that leads to the hypersensitivity response^ 26,27^. PAW treatment also induced the expression of *CrSGD*, a key enzyme in the alkaloid’s biosynthetic pathway, involved in the plant defence responses. This gene plays an important role in the upstream regulation of the pathway leading to the production of vincristine and vinblastine, having antimicrobial activity against phytopathogenic fungi and bacteria^ 28^. Its level constantly increased from 24 to 96 h pt, with an overexpression of ca. 5, 5.4 and 6.8-fold respectively. Its activation can also be due to the H_2_O_2_ as reported by Tang et al. ^29^ who showed that different concentrations of exogenous H_2_O_2_ increased the concentration of alkaloids including vinblastine, vindoline and catharanthine.

### Resveratrol producing pathway is induced in PAW treated grapevine plants

**Figure 2:**
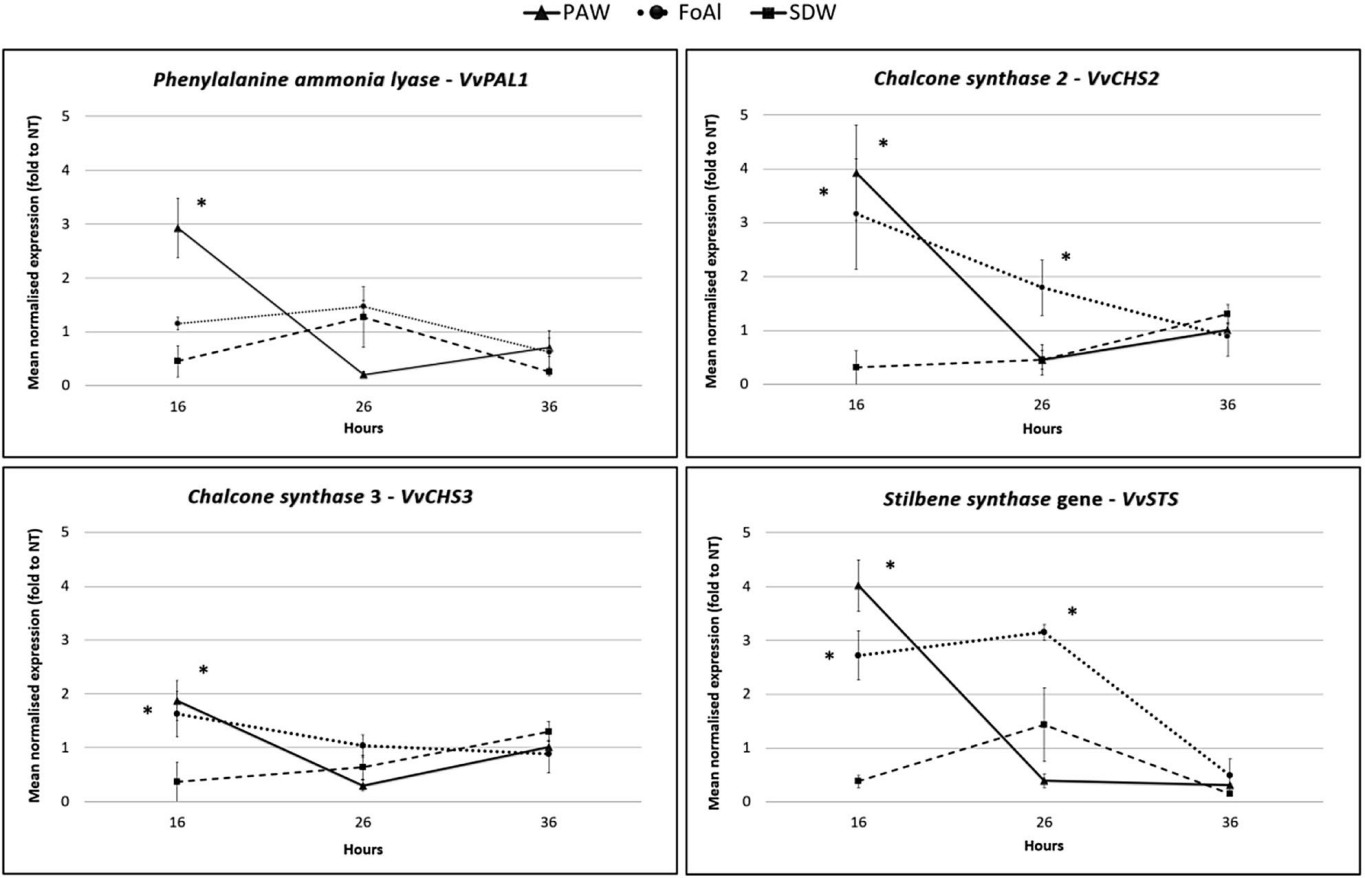
Changes in grapevine gene expression after PAW treatment. Relative expression of phenylalanine ammonia lyase *VvPAL1*, chalcone synthase 2 *VvCHS2*, chalcone synthase 3 *VvCHS3* and stilbene synthase *VvSTS* determined by qRT-PCR after PAW, SDW and FoAl treatments. Their relative expression (actin, ubiquitin and glyceraldehydes 3-phosphate dehydrogenase were internal controls) to untreated shoots are shown at different time points after treatment. *Statistically significant *P* < 0.05.

PAW’s activity in grapevine plants cv Chardonnay showed an increased expression of *VvPAL1*, *VvCHS2*, *VvCHS3* and *VvSTS* genes at 16 h pt. Furthermore, the relative expression of the *VvPAL1* gene resulted 2.9 fold higher than untreated controls while the *VvCHS2*, *VvCHS3* and *VvSTS* expression increased by 3.9, 1.9 and four fold, respectively (Fig. 2). Moreover, *VvCHS1* didn’t show any variation (Supplementary File 2). The *VvPAL1* gene encodes the enzyme producing the precursor of shikimate and is involved in the phenylpropanoid metabolic pathways^30^ through the formation of resveratrol (catalyzed by stilbene synthase, STS) or flavonoids (catalyzed by chalcone synthase, CHS) ^31^. These metabolic pathways have been extensively studied in grapevine as they contribute to the pigmentation of flowers, fruits, seeds and leaves and are involved in different physiological and biochemical processes ^32^. Resveratrol and its derivatives such as 3- and a-viniferin, pterostilbene and piceatonol represent the main phytoalexins in grapevine ^33^. These molecules are synthesized in grapevine also in response to *Erysiphe necator*, *Plasmopara viticola* and *Botrytis cinerea* infections^ 34–36^. In spite of differences registered in the expression of some genes, the overall results obtained confirmed the positive effect of PAW treatment on the phytoalexin pathway, in both, micropropagated periwinkle shoots and grapevine plants. On the other hand, the effect of fosetyl aluminium (FoAl), used as control, was more variable. Moreover, preliminary tests carried out with a synthetic solution with the same concentration of hydrogen peroxide and nitrates and the same pH of PAW, showed the same behaviour of SDW negative control (data not shown), confirming that PAW effect is due to a complex of factors^ 13^.

### PAW induced changes in the expression of periwinkle miRNAs regulating the defence gene expression

Six small RNA (sRNA) libraries were constructed using total RNAs from SDW treated control (H_2_O1, H_2_O2, H_2_O3) and PAW-treated periwinkle plants (PAW1, PAW2, PAW3). Small RNA sequencing yielded 18,862,867 and 21,163,320 high-quality raw reads from the H_2_O and PAW treated plant libraries, respectively (Supplementary File 3). The length distribution of the total reads revealed that the majority of them from each library was ranging 20–25 nt: those of 24-nt were the most abundant, followed by the 21-nt reads. Overall, 81 microRNAs (miRNAs) belonging to 36 families were showing significant changes after the PAW treatment, among them miR166, miR159 and miR396 families were the most represented. In particular, miRNA belonging to miR157, miR172, miR393, miR5368 and miR8016 families were found down-regulated, while miR159, miR165, miR319, miR395, miR396, miR398 and miR399 families were up-regulated after the PAW treatment (Table 2).

**Table 2:** miRNAs showing significantly differential expression in PAW-treated periwinkle plants: those down-regulated are listed on the left and the up-regulated on the right of the table.

miRNA	PAW	SDW	miRNA	PAW	SDW
	Normalized reading^a^			Normalized reading^a^	
miR157a	32,45	76,13	miR159a	34.526,29	22.389,27
miR157d-3p	0,76	10,49	miR165a	223,37	148,96
miR166d	1.188,44	1.726,98	miR166f	1.021,06	710,88
miR172a*	18,99	43,88	miR319a	613,36	302,02
miR172c-5p	3,47	8,13	miR395a	15,64	0,77
miR172c	6,69	25,92	miR396a-3p*	789,64	477,53
miR393a	128,95	331,73	miR396a*	9.791,12	3.473,35
miR5368	2,25	14,79	miR396b	7.084,25	5.558,85
miR8016	89,19	273,77	miR398b	24.470,24	18.283,28
novel-79	31	47,96	miR399b	33,6	21,05
miRnovel05	276,79	327,54			

^a^Average reading of three biological replicates.

**Figure 3:**
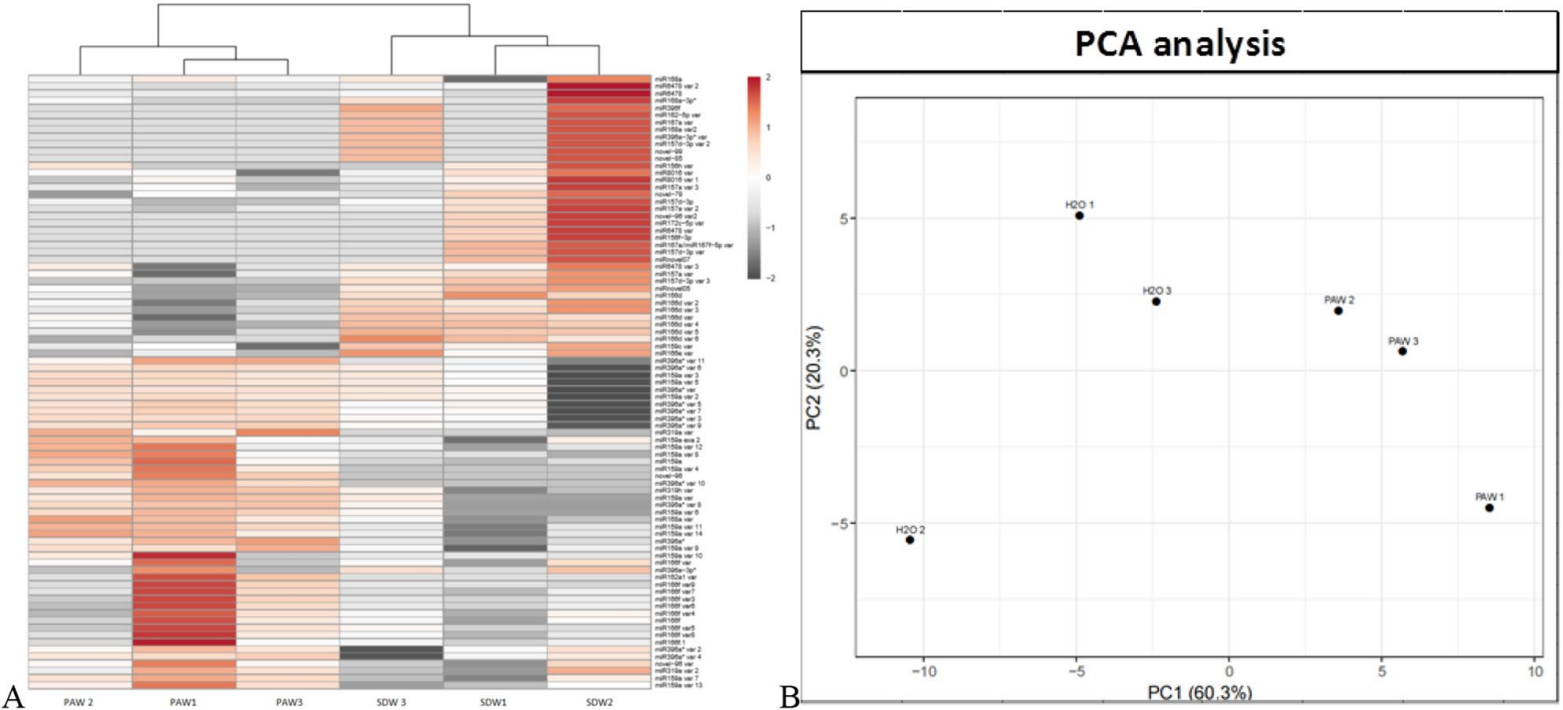
(**A**): Heat-map of the log2-fold change differentially expressed miRNAs and (**B**) principal component analysis (PCA) score plot of the miRNA expression in PAW- (PAW1, PAW2, PAW3) and SDW- (H_2_O1, H_2_O2, H_2_O3) treated periwinkle plants.

The heat map shows two distinct expression clusters differentiating the PAW- treated and untreated libraries, the results of the treatment were statistically similar in the three biological replicates (Fig. 3).

**Figure 4:**
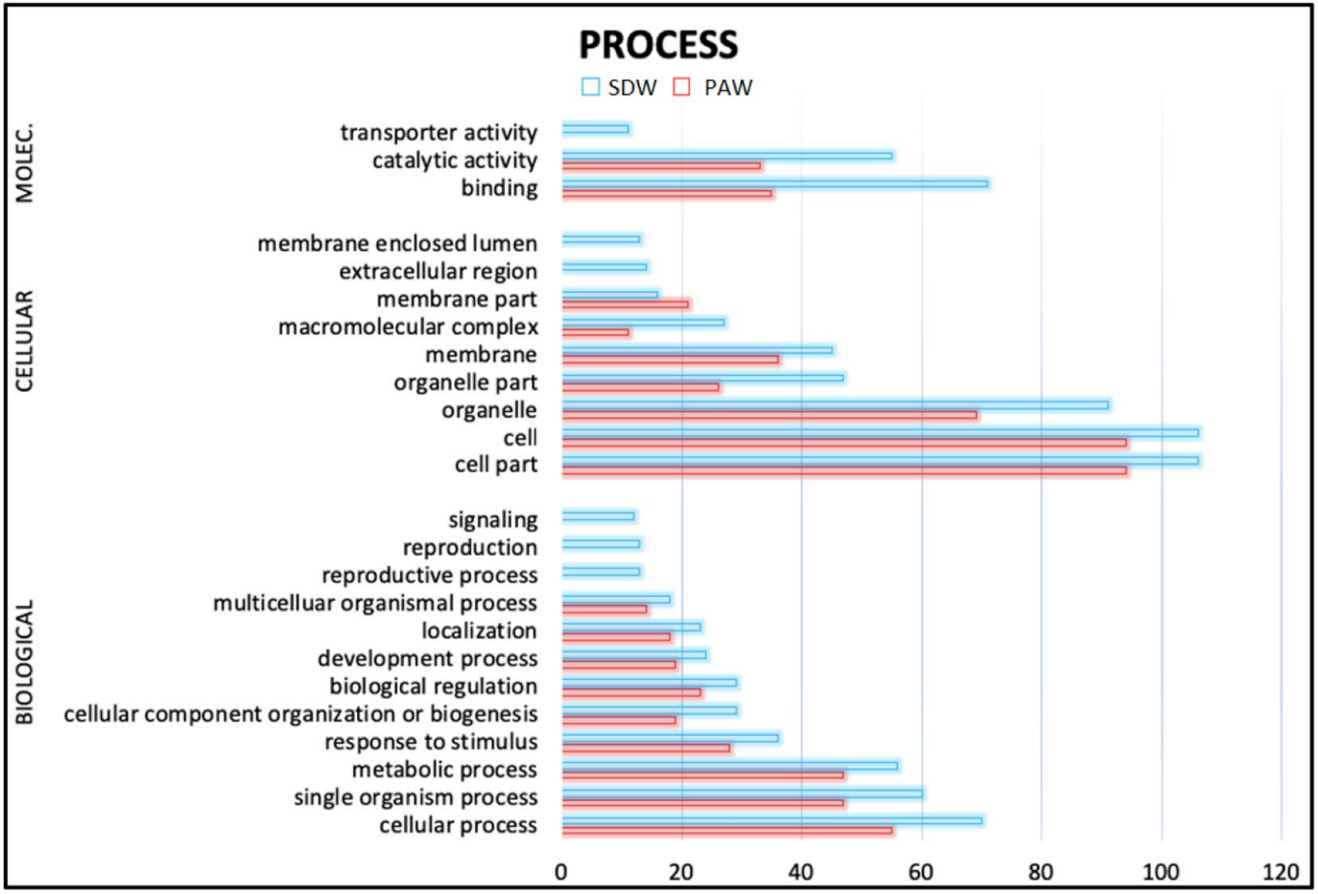
GO enrichment of miRNA target genes for which the expression level was increased or decreased by the PAW treatment compared with the SDW treatment. The dataset containing protein sequences of *C. roseus* genome was set as background. The vertical axis corresponds to the enriched GO category, and the horizontal axis to the GO enrichment.

To investigate the relevance of the changes in the miRNA pattern the possible targets of the *Catharanthus roseus* miRNAs were predicted and putative target genes were analyzed by GOterms with the aid of the Blast2GO program with default parameters. Genes with a known function were categorized by biological process, molecular function, and cellular component according to the ontological definitions of the GOterms (Fig. 4).

Among the miRNAs overexpressed in the PAW-treated plants miR159, miR395 and miR398 families were reported as up-regulated under H_2_O_2_ exposition^37^. Induced expression of miR165 and miR166f families is targeting 2 serine/threonine phosphatase 2C proteins (ABI1 and ABI2), two key enzymes usually inactivated by high concentrations of GSH (glutathione), in presence of H_2_O_2_ accumulation in the plant cells^38^ (Supplementary File 4). The up-regulation of a miRNA usually results in the degradation of its target gene in plants; furthermore the target genes of up-regulated miRNAs may be associated with stress response, whereas the target genes of down regulated miRNAs are usually involved in stress resistance. The over expression of these miRNAs, together with the lower expression of miR157, a negative regulator of GST (glutathione-s-transferase, key enzyme in oxidative response^25^, confirms the oxidative response activation by the PAW treatment. The miR157, whose target genes are involved in the actin and myosin microtubules formation, was down-regulated in the PAW-treated plants. This result supports the finding of an over expression of callose synthase gene, leading to a callose accumulation in the cell, in the PAW-treated shoots. The miR398 targets three superoxide dismutase genes* CSD1*, * CSD2* and *CSD3*, key enzymes in the ROS detoxification. Its overexpression in the PAW-treated plants could reduce the transcription level of these genes and therefore reduce plant's ability to degrade the oxidative compounds. In particular the down-regulation of these genes could reduce the H_2_O_2_ degradation and increase the signal defence transduction^39^. Alternatively it could downstream regulate the genes activated for the detoxification of ROS species both, contained in PAW solution and produced by plant in response to the treatment. Moreover, among the miRNA families over expressed in the PAW-treated plants, the miR399 and miR395 families are involved in down regulation of some genes under nutritional deficiencies, in order to enhance the activation of nitrogen, phosphate and sulphate absorption ^40^; this could be an interesting aspect to be developed for future application in plant fertilization strategies.

## Conclusions

The exposure of SDW to a nanosecond pulsed DBD enables the production of PAW characterized by the presence of oxygen and nitrogen reactive species (ROS and RNS), that are the main molecules produced in plants under stress. Transcriptional (qRT-PCR) and post-transcriptional (miRNA) molecular analyses highlighted PAW’s ability to enhance the expression of genes encoding the main enzymes involved in the phytoalexin biosynthetic pathway (alkaloids for *C. roseus*, and stilbenes for *V. vinifera*) and to modulate some of the stress response genes through miRNAs regulation. The results obtained provide an encouraging framework for applying PAW technology in plant disease management programs. PAW could be applied under greenhouse conditions to increase the yields and crop quality reducing the water usage in an economic and environmentally sustainable manner. Future studies will focus on the scale up of the system and on the further identification of PAW components inducing the resistance in plants, in order to maximize its concentration and thus its effect.

## Methods

### PAW production

**Figure 5:**
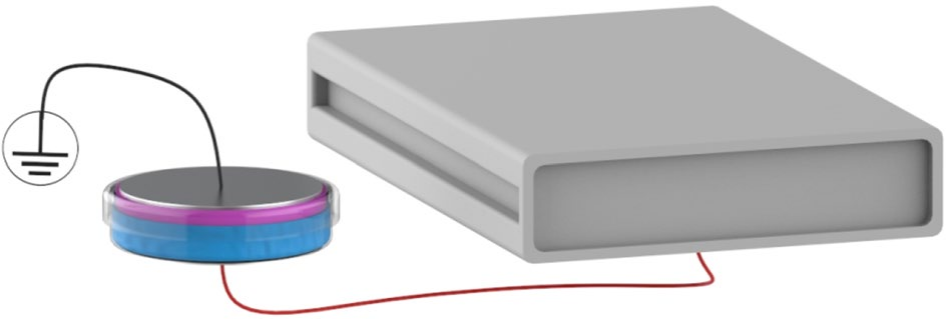
Schematic picture of the setup implemented for the production of PAW.

A total of 80 mL of SDW was exposed to a nanosecond pulsed dielectric barrier discharge (Fig. 5), operating in ambient air for a 10 min treatment, with a peak voltage of 19 kV and a pulse repetition frequency of 1 kHz, as reported^15^. Immediately after the treatment the PAW aliquots produced were frozen. The day after production, PAW was melt at room temperature, pH was monitored and concentrations of hydrogen peroxide and nitrates induced by plasma treatment were measured using the Amplex Red Hydrogen Peroxide Assay Kit (Thermo Fisher Scientific, USA), and the nitrate/nitrite colorimetric assay (ROCHE, Switzerland), respectively. The measurement of these reactive species was performed according to the manufacturer’s protocols. The absorbances were measured photometrically with a microplate reader (Rayto, P.R. China).

### Plant materials and growth conditions

Healthy periwinkle shoots [*Catharanthus roseus* (G) Don] were micropropagated from one mother plant with white flowers and maintained in vitro^40^ in a phytotron under controlled conditions (24 ± 2 °C and 16 h day light). Healthy periwinkle plants derived from the ones in micropropagation and healthy grapevine plants cv Chardonnay were soil grown in a greenhouse under insect proof conditions. The greenhouse conditions were set as 16 h light at 30 °C and 8 h dark at 24 °C, and the RH (relative humidity) was maintained at 70–75% until the end of the experiment.

### PAW treatments

**Figure 6:**
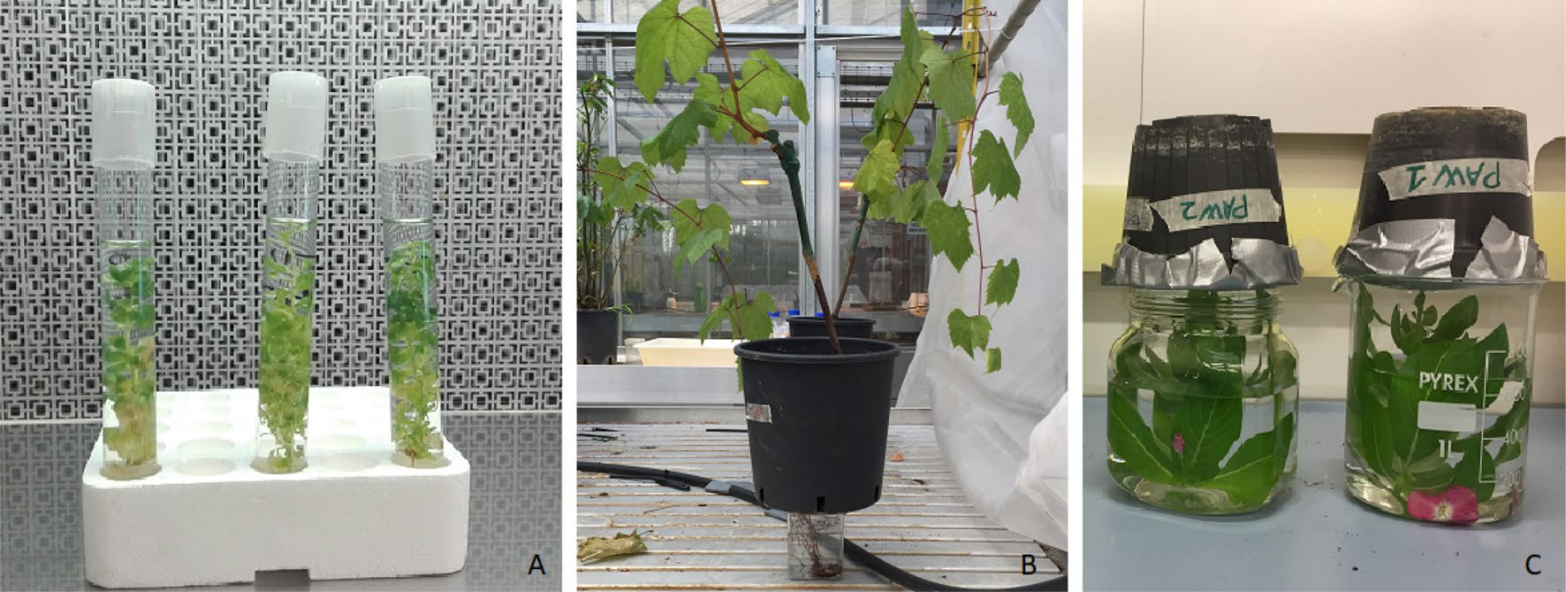
PAW treatments. (**A**) Micropropagated periwinkle shoots treated by submersion; (**B**) grapevine plants cv. Chardonnay treated by root drenching; (**C**) periwinkle plants treated by upside down submersion.

To longer maintain PAW's physical and chemical properties, different treatments were employed according with the plant material (Fig. 6). Periwinkle shoots (5 replicates/treatment) were submersed in the micropropagation glass tube for 25 min with 20 mL PAW and SDW (sterile distilled water, negative control). A solution of 2.5 g/L fosetyl aluminium (Aliette, Bayer Crop Science, Italy) was then used as control, due to its known capacity to stimulate the production of pathogenesis-related (PR) proteins^41^. Additional control was represented by a synthetic solution prepared as following: SDW was supplemented with 13.5 mg × L^−1^ of H_2_O_2_ (Sigma-Aldrich) and 81.9 mg × L^−1^ of NO_3_^−^, the same concentrations generated by plasma treatment of SDW. Moreover HCl has been used to adjust the pH, to have a final solution with same pH and concentration of H_2_O_2_ and NO_3_^−^ of PAW. This solution has been used for the treatment of plant and did not induce any effect on them. The entire shoots were collected from each replicate/treatment at six time points: 0 h, 7 h, 24 h, 48 h, 96 h, and 120 h. Four untreated shoots at each time point were used as negative control and calibrator for data normalisation. The tissues were flash frozen in liquid nitrogen and stored at − 80 °C until RNA extraction.

Under greenhouse conditions, 2-year old grapevine plants (3 plants/treatment) were partially uprooted, the roots were washed and irrigation devices were applied to acclimatize the plants before the treatments. After 3 weeks the root apparatus was drenched for 25 min into 450 mL of PAW, SDW, FoAl (the same three treatments described above) and the 4th, 5th and 6th leaf of each plant/treatment was collected at 3 time points: 16 h, 26 h, 36 h. Three untreated shoots at each time point were used as negative control and calibrator for data normalisation. The tissues were flash frozen in liquid nitrogen and stored at − 80  °C until RNA extraction.

For small RNA high-throughput sequencing (HTS) periwinkle plants at the 5th–6th leaf stage (3 replicates/treatment) were immersed upside down for 25 min in a 650 mL of PAW and SDW. The entire plants were collected after 16 h and flash frozen in liquid nitrogen and stored at − 80  °C until RNA extraction.

### RNA extraction from different plant sources

From micropropagated periwinkle shoots total RNA was extracted from 100 mg of material using Qiagen RNeasy Plant Minikit (# 74,904) in combination with RNase-Free DNase Set (Qiagen # 79,254) following the instructions of the manufacturer. The total RNA, eluted in 50 μL of nuclease free distilled water, was stored at − 20 °C.

Total RNA from grapevine plants was extracted as described^42^. Samples (200 mg), powdered in liquid nitrogen, were added to 900 μL of extraction buffer (2% CTAB, 2.5% PVP-40, 2 M NaCl, 100 mM Tris–HCl pH 8.0, 25 mM EDTA pH 8.0) and 2% of β-mercaptoethanol added just before use. The buffer was previously heated at 65 °C and incubated for 10 min. An equal volume of chloroform: isoamyl alcohol (24: 1 v/v) was added and the tube was inverted vigorously, and centrifuged at 11,000×*g* for 10 min at 4 °C. The supernatant was recovered and re-extracted with chloroform/ isoamyl alcohol. The supernatant, in a new microcentrifuge tube, was added with 0.25 volumes of LiCl 3 M. The mixture was incubated in ice for 30 min and the RNA was selectively pelleted after centrifugation at 21,000×*g* for 20 min at 4 °C. The pellet was resuspended in 500 μL of SSTE buffer (10 mM Tris–HCl pH 8.0, 1 mM EDTA pH 8.0, 1% SDS, 1 M NaCl) pre-heated at 65 °C. An equal volume of chloroform/ isoamyl alcohol was added and the mixture was centrifuged at 11,000 g for 10 min at 4 °C. From the supernatant in a new microcentrifuge tube the RNA was precipitated with 0.7 volumes of cold isopropanol and immediately centrifuged at 21,000×*g* for 15 min at 4 °C. The pellet was washed with ethanol (70%), dried, resuspended in 50 μL of nuclease free distilled water, and stored at − 20 °C.

For small RNA HTS total nucleic acid was extracted from periwinkle plants using a phenol–chloroform method ^42^. The samples (200 mg), powdered in liquid nitrogen, were added to 600 μL extraction buffer [2% SDS, 1 × extraction buffer (1 M glycine, 100 mM EDTA, 1 M NaCl), pH 9.5] and to an equal volume of phenol. After a centrifugation at 15.000 rpm for 5 min, the aqueous phase was added to 600 μL phenol/chloroform (1:1) and centrifuged under the same conditions. The aqueous phase was added to 600 μL of chloroform, centrifuged as above and added to 0.1 volumes of NaOAc (CH_3_COONa) and to 2.2 volumes of absolute cold ethanol. Tubes were mixed by inversion and stored 10 min in ice before a centrifugation at 15.000 rpm at 4 °C for 30 min. The pellet was washed with ethanol (70%), dried and resuspended in 50 μL of nuclease free distilled water and stored at − 20 °C. Four RNA extractions, 2 from stems and 2 from leaves, were carried out from each plant. Small RNA sequencing libraries were prepared from the pool of these RNAs as described below. Quantity and quality (ratio A260 nm/A280 nm) of each RNA extract were measured using the Nanodrop 1000 (ThermoScientific Fisher, USA). The quality was also verified by loading 5 μL of RNA extracts into a 1% agarose gel followed by electrophoresis at 130 V for 100 min in TAE buffer. The gel was stained in ethidium bromide solution (0.03%) for 20 min and destained in distilled water for 5 min; visualisation was then carried out under UV light (312 nm). All RNA samples were diluted at 250 ng/μL before the molecular assays.

### Reverse transcription quantitative polymerase chain reaction (RT-qPCR) analysis

M-MLV reverse transcriptase (RT, Promega, USA) was employed to synthesize cDNAs using random hexamer primer (Fermentas, Lithuania) and following the manufacturer’s instructions. For all genes of interest, ~ 1.5 ng of cDNA template was used for qPCR, with the expression normalized to the ubiquitin gene (periwinkle) and to actin, ubiquitin and glyceraldehydes 3-phosphate dehydrogenase genes (grapevine). All the qPCR reactions were performed using the SYBR Green master mix (Applied Biosystems, Foster City, CA, USA) performing three technical replicates, and a minimum of three biological replicates per experiment. Specific primers for RT-qPCR in periwinkle shoots/plants and grapevines were designed by using Primer Blast (https://www.ncbi.nlm.nih.gov/tools/primer-blast/) software and checked by Amplify 3 ^43^. The target genes selected for periwinkle samples were the callose synthase (*CrCalS11*), important in defence reaction, and the phenylalanine ammonium lyase (*CrPAL1*), strictosidine-β-glucosidase (*CrSGD*), deacetylvindoline-O-acetyltransferase (*CrDAT*) and one chalcone synthase (*CrCHS*), genes involved in the phytoalexin pathway. For the grapevine samples the genes selected were the phenylalanine ammonium lyase (*VvPAL1*), the chalcone synthases 2 and 3 (*VvCHS2 *and *VvCHS3*) and the stilbene synthase (*VvSTS*). The primers used for RT-qPCR and the accession numbers of the gene sequences are listed in the Supplementary File 5. The RT-qPCR assay was performed using RealTime PCR ABIPRISM StepOne sequence detection system (Applied Biosystem, Foster City, CA, USA). The primers specificity was evaluated on RNA extracts by a dissociation curve analysis in order to exclude non-specific amplifications or primer dimers presence. The thermal profile was set up as follows: 95 °C for 10 min, 40 cycles at 95 °C for 15 s, and 60 °C for 1 min. Dissociation curves were performed at 95 °C for 15 s, 60 °C for 30 s and 95 °C for 15 s. The efficiency of each primer pair was determined using LinRegPCR software^44^. All gene transcription levels were reported as Mean Normalised Transcription relative to the genes employed as reference gene using the equation 2^−ΔCT^, where ΔCT is CT target gene–CT reference gene (for periwinkle samples) or geometric mean of the three reference genes (for grapevine samples). The mean data obtained were analyzed by ANOVA (*P* 0.05) followed by Student test ^45^.

### Preparation of small RNA sequencing libraries and bioinformatics analysis of the sequenced reads

Six small RNA sequencing libraries (3 from PAW- and 3 from SDW-treated periwinkle samples) were prepared using Baggerley’s test. Statistically significant differential expressed miRNAs were selected using FDR p value correction small RNA fraction was purified on polyacrylamide gel. Adapters were ligated and cDNA was synthesised, and PCR amplified. The product was gel-purified and sequenced on Illumina using HiSeq2000 sRNA-seq platform. Fastq files with the raw reads are deposited into NCBI GEO under the code GSE146177.

Adapters from the sequenced reads were trimmed with CLC Genomics Workbench (v11.0, CLCbio, Arhus, Denmark) and only the good quality reads between 16–28 nt were kept. Small RNAs were also mapped to the *C. roseus* transcriptome (https://nipgr.res.in/mjain.html?page=catharantus) allowing one mismatch during the analysis, and the relative expression of the small RNAs was counted and normalized to 1 million sequenced reads. Results of the biological replicates were compared using the same software as above and statistical differences were then analysed by Baggerley’s test. Statistically significant differential expressed miRNAs were selected using FDR *P* value correction < 0.05 ^46^ and the values were expressed in logarithmic function (log_2_). Further statistical analyses were carried out by a distributional fold change (DFC) test ^47^ and by IQR index (interquartile range)^ 48^, selecting only miRNAs differences > 2 and > 5, respectively. The differences obtained by statistical analyses data were processed by Clusvis web software ^49^ and heat maps were produced.

### Target prediction for the differentially expressed miRNAs

Target genes were firstly predicted using *C. roseus* transcriptome, then the results were confirmed by psRNA Target online software (https://www.plantgrn.org/psRNATarget/)^50^ combined with CLC Genomics Workbench (v 11.0, CLCbio Arhus, Denmark) and Blast2Go^51^ programs. Only the shared predictions of the three softwares were considered as target genes. The biological processes, molecular functions, and cellular components of these genes were examined using the Blast2Go to perform Gene Ontology (GO) annotation and GO enrichment analysis. The statistical test method was set as Fisher and the multi-test adjustment method was set as Bonferroni. The threshold of significance was defined as *P* < 0.01 and the false discovery rate (FDR) was defined as < 0.01.

## Supplementary information

Supplementary File 1.

Supplementary File 2.

Supplementary File 3.

Supplementary File 4.

Supplementary File 5.
