# Supplementary material for: Plasma activated water triggers plant defence responses
Source: Sci Rep. 2020 Nov 5;10:19211. doi: 10.1038/s41598-020-76247-3 (PMC7644721; doi:10.1038/s41598-020-76247-3)
Supplement: Supplementary file 1 [file 41598_2020_76247_MOESM1_ESM.doc]

**Title: Plasma activated water triggers plant defence responses**

**Journal: Scientific Reports**

Yuri Zambon^1^, Nicoletta Contaldo^1*^, Romolo Laurita^2^, Eva Várallyay^3^, Alessandro Canel^1^, Matteo Gherardi^2,4^, Vittorio Colombo^2,4^, Assunta Bertaccini^1^

***corresponding author:** [**nicoletta.contaldo2@unibo.it**](mailto:nicoletta.contaldo2@unibo.it)

^1^Department of Agricultural and Food Sciences (DISTAL), Plant Pathology, *Alma Mater Studiorum*- University of Bologna, V. le Fanin, 40, Bologna, 40127, Italy

**
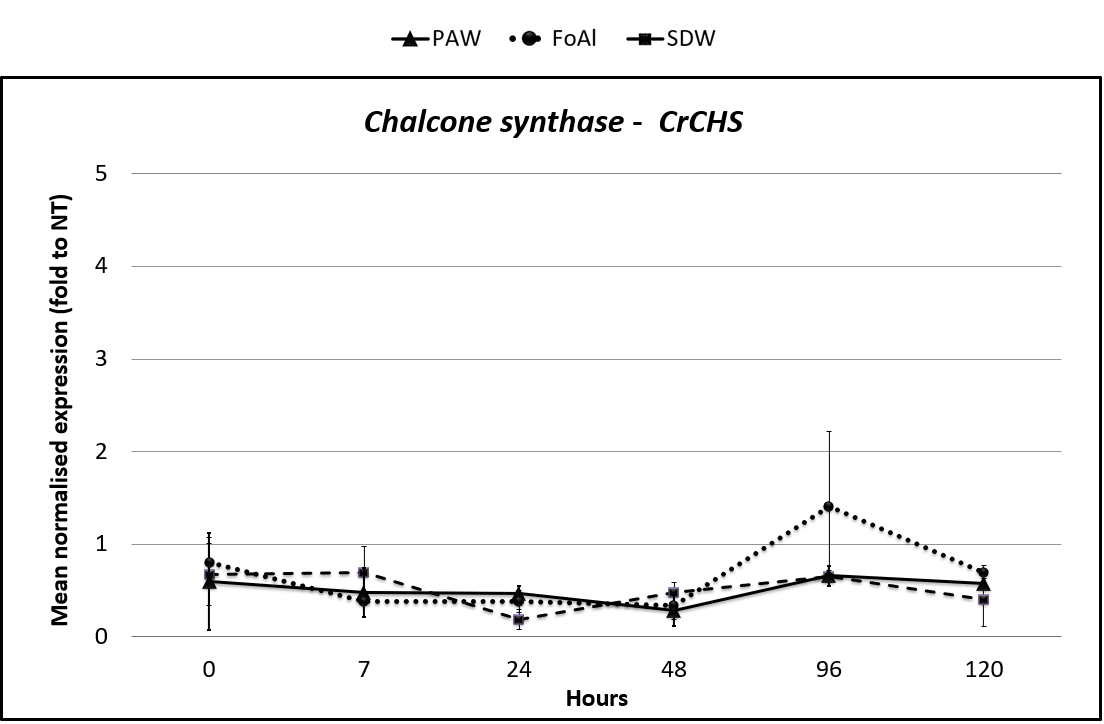
**

**Supplemetary file 1 Micropropagated periwinkle shoots gene expression**. Chalcone synthase expression kinetics in micropropagated periwinkle shoots after PAW, FoAl and SDW treatments. The graph shows the gene induction at each time point and the standard error (±SE)
