# Supplementary material for: Plasma activated water triggers plant defence responses
Source: Sci Rep. 2020 Nov 5;10:19211. doi: 10.1038/s41598-020-76247-3 (PMC7644721; doi:10.1038/s41598-020-76247-3)
Supplement: Supplementary file 3 [file 41598_2020_76247_MOESM3_ESM.doc]

**Title: Plasma activated water triggers plant defence responses**

**Journal: Scientific Reports**

Yuri Zambon^1^, Nicoletta Contaldo^1*^, Romolo Laurita^2^, Eva Várallyay^3^, Alessandro Canel^1^, Matteo Gherardi^2,4^, Vittorio Colombo^2,4^, Assunta Bertaccini^1^

***corresponding author:** [**nicoletta.contaldo2@unibo.it**](mailto:nicoletta.contaldo2@unibo.it)

^1^Department of Agricultural and Food Sciences (DISTAL), Plant Pathology, *Alma Mater Studiorum*- University of Bologna, V. le Fanin, 40, Bologna, 40127, Italy

| **Thesis** | **Libraries** | **Readings** | **Reading without adapters** |
| --- | --- | --- | --- |
| PAW | Biological replication 1 | 26.901.619 | 26.607.758 |
|  | Biological replication 2 | 23.315.061 | 23.133.444 |
|  | Biological replication 3 | 13.273.280 | 12.719.647 |
| H_2_O | Biological replication 1 | 25.353.587 | 25.028.886 |
|  | Biological replication 2 | 17.344.132 | 16.902.029 |
|  | Biological replication 3 | 13.890.882 | 13.660.161 |
|  |  |  |  |

**Supplementary File 3** Total small RNA (sRNA) reads obtained for each biological replication
