# Supplementary material for: Plasma activated water triggers plant defence responses
Source: Sci Rep. 2020 Nov 5;10:19211. doi: 10.1038/s41598-020-76247-3 (PMC7644721; doi:10.1038/s41598-020-76247-3)
Supplement: Supplementary file 4 [file 41598_2020_76247_MOESM4_ESM.doc]

**Title: Plasma activated water triggers plant defence responses**

**Journal: Scientific Reports**

Yuri Zambon^1^, Nicoletta Contaldo^1*^, Romolo Laurita^2^, Eva Várallyay^3^, Alessandro Canel^1^, Matteo Gherardi^2,4^, Vittorio Colombo^2,4^, Assunta Bertaccini^1^

***corresponding author:** [**nicoletta.contaldo2@unibo.it**](mailto:nicoletta.contaldo2@unibo.it)

^1^Department of Agricultural and Food Sciences (DISTAL), Plant Pathology, *Alma Mater Studiorum*- University of Bologna, V. le Fanin, 40, Bologna, 40127, Italy

| **miRNA** | **Gene** | **Protein** | **Target description** |
| --- | --- | --- | --- |
| **miR166 (166f)** | RDR6 | RNA-dependent RNA polymerase 6 | defense response to virus/ gene silencing by RNA |
|  |  |  | maintenance of shoot apical meristem identity |
|  |  |  | production siRNA involved in RNA interference |
|  | ATHB-14 | Homeobox-leucine zipper protein ATHB-14 | adaxial/abaxial pattern specification |
|  |  |  | primary shoot apical meristem specification |
|  | A G O 10 | Protein argonaute 10 | defense response to virus/ gene silencing by RNA |
|  |  |  | miRNA metabolic process |
|  |  |  | multicellular organism development |
| **miR159a** | MYB33 | Transcription factor MYB33 | gibberellic acid mediated signaling pathway |
|  |  |  | negative regulation of cell proliferation |
|  |  |  | + regulation of ABA-activated signaling pathway |
|  |  |  | + regulation of programmed cell death |
|  |  |  | protein storage vacuole organization |
|  |  |  | response to cytokinin, ethylene and giberellin |
| **miR319a** | TCP4 | Transcription factor TCP4 | cell differentiation |
|  |  |  | cotyledon morphogenesis |
|  |  |  | + regulation of development, heterochronic |
|  | LOX 2 | Lipoxygenase 2, chloroplastic | green leaf volatile biosynthetic process |
|  |  |  | jasmonic acid biosynthetic process |
|  |  |  | lipid oxidation - oxylipin biosynthetic process |
| **miR395a** | OsAPx7 - SIF3HL- | Stromal Ascorbate Peroxidase - Flavonone 3 hydroxylase - LRR protein | ATP sulphurylases |
|  |  |  | sulphur trasporters |
|  |  |  | Stromal ascorbate peroxidase |
|  |  |  | flavanone3-hydroxylase |
|  |  |  | Disease resistance family protein/LRR protein |
| **miR399b** | UBC24 | Probable ubiquitin-conjugating enzyme E2 24 | cellular protein catabolic process |
|  |  |  | cellular response to phosphate starvation |
|  |  |  | regulation of phosphate transmembrane transport |
|  | PHR1 | Protein phosphate starvation response 1 | cellular response to high light intensity |
|  |  |  | cellular response to phosphate starvation |
|  |  |  | circadian rhythm |
|  |  |  | sulfate ion homeostasis |
| **miR398b** | CSD2 | Superoxide dismutase [Cu-Zn] 2, chloroplastic | cellular response to light intensity |
|  |  |  | cellular response to oxidative stress, ozone |
|  | CCS | Copper chaperone for superoxide dismutase, chloroplastic/cytosolic | cellular copper ion homeostasis |
|  |  |  | metal ion tran sport |
| **miR165a** |  | Transcription factor Phabulosa-Phavolita | HD-Zip trascription factors (Phabulosa-Phavolita) |
|  |  |  | ABA regulation with mir166 |
|  |  |  | serine/threoninephosphatase7 |
| **miR172**  **(172a-172c)** | GI | Protein GIGANTEA APETALA | cell differentiation |
|  |  |  | circadian rhythm |
|  |  |  | flower development |
|  |  |  | positive regulation of long-day photoperiodism, flowering |
|  |  |  | response to light |
|  |  |  | response to cold |
|  |  |  | esponse to hydrogen peroxide |
|  | TOE2 | AP2-like ethylene-responsive transcription factor TOE2 | ethylene-activated signaling pathway |
|  |  |  | multicellular organism development |
|  | RAP2-7 | Ethylene-responsive transcription factor RAP2-7 | basichelix-loop-helix (bHLH) DNA-binding superfamily protein |
|  | TOE3 | Trascription factor TOE3 | microtubule associated complex |
| **mir157a - miR157d-3p** | SPL4 | Squamosa promoter-binding-like protein 4 | flower development |
|  |  |  | egulation of vegetative phase change |
|  |  |  | cell wall pectin metabolic process |
|  |  |  | Tubulin family proteins |
|  | ARP | Actin related protein | actin-relatedprotein |
|  |  |  | phospholipase A2A |
|  |  |  | glutathioneS-transferase family protein |
|  |  |  | glutathione S-transferaseTAU16 |
| **miR393a** | TIR1 | Protein transport inhibitor response 1 | auxin-activated signaling pathway |
|  |  |  | cell cycle |
|  |  |  | defense response |
|  |  |  | ethylene-activated signaling pathway |
|  |  |  | stamen development |
| **miR166 (166d)** | RDR6 | RNA-dependent RNA polymerase 6 | defense response to virus |
|  |  |  | gene silencing by RNA |
|  |  |  | maintenance of shoot apical meristem identity |
|  |  |  | production of siRNA involved in RNA interference |
|  | AGO10 | Protein argonaute 10 | gene silencing by RNA |
|  |  |  | miRNA metabolic process |
|  |  |  | multicellular organism development |
| **miR8016** |  |  | Protein phosphatase 2C family protein |
|  |  |  | response to other organism |

**Supplementary File 4** Potential targets of miRNAs differentially expressed in *C. roseus* after PAW treatment
