# Supplementary material for: Plasma activated water triggers plant defence responses
Source: Sci Rep. 2020 Nov 5;10:19211. doi: 10.1038/s41598-020-76247-3 (PMC7644721; doi:10.1038/s41598-020-76247-3)
Supplement: Supplementary file 5 [file 41598_2020_76247_MOESM5_ESM.doc]

**Title: Plasma activated water triggers plant defence responses**

**Journal: Scientific Reports**

Yuri Zambon^1^, Nicoletta Contaldo^1*^, Romolo Laurita^2^, Eva Várallyay^3^, Alessandro Canel^1^, Matteo Gherardi^2,4^, Vittorio Colombo^2,4^, Assunta Bertaccini^1^

***corresponding author:** [**nicoletta.contaldo2@unibo.it**](mailto:nicoletta.contaldo2@unibo.it)

^1^Department of Agricultural and Food Sciences (DISTAL), Plant Pathology, *Alma Mater Studiorum*- University of Bologna, V. le Fanin, 40, Bologna, 40127, Italy

| **Gene** | **Primer** | **Sequence 5'-3'** | **GenBank a.n.** | **Literature** |
| --- | --- | --- | --- | --- |
| *CrPAL1* ^a^ | Forward | AAACCCGGCAATCCCTAACC | AB042520 | [this study] |
|  | Reverse | GGAATTCGGCTCCCACATC |  |  |
| *[CrCHS* ^a^ | Forward | ACTCCTCCGGCTACCTAC | AJ131813 | [this study] |
|  | Reverse | TCCTTGAGCACGTTGAGC |  |  |
| *CrSGD* ^a^ | Forward | TCACAAAGCTGCTGTGGAAG | AF112888 | [52] |
|  | Reverse | CACCCGTTGTTAATGGCTCT |  |  |
| *CrDAT* ^a^ | Forward | CCTCCGGAAGCCATAGAAAAG | LN809931 | [this study] |
|  | Reverse | CGTGGCACATCGACTGAGAA |  |  |
| *CrCalS11* ^a^ | Forward | GCTGCTTATGCGCTCGACTATG | HM800500 | [53] |
|  | Reverse | GTATCGTCTCTCGGCAACCCAC |  |  |
| *CrUBQ11* ^a^ | Forward | GGAAGGCATTCCACCAGACCA | EU007433 | [54] |
|  | Reverse | TACCTCCCCGGAGACGAAGC |  |  |
| *VvPAL1* ^b^ | Forward | TGCTGACTGGTGAAAAGGTG | X75967 | [55] |
|  | Reverse | CGTTCCAAGCACTGAGACAA |  |  |
| *VvCHS1* ^b^ | Forward | AGCCAGTGAAGCAGGTAGCC | AB015872 | [58] |
|  | Reverse | GTGATCCGGAAGTAGTAAT |  |  |
| *VvCHS2* ^b^ | Forward | TCTGAGCGAGTATGGGAACA | AB066275 | [58] |
|  | Reverse | AGGGTAGCTGCGTAGGTTGG |  |  |
| *VvCHS3* ^b^ | Forward | GTTTCGGACCAGGGCTCACT | AB066274 | [58] |
|  | Reverse | GGCAAGTAAAGTGGAAACAG |  |  |
| *VvSTS* ^b^ | Forward | GTGGGGCTCACCTTTCATT | AF274281 | [55] |
|  | Reverse | CTGGGTGAGCAATCCAAAAT |  |  |
| *VvACT* ^b^ | Forward | TCAGCACTTTCCAGCAGATG | TC30205 | [55] |
|  | Reverse | TAGGGCAGGGCTTTCTTTCT |  |  |
| *VvUBQ1* ^b^ | Forward | GTGGTATTATTGAGCCATCCTT | TC32075 | [56] |
|  | Reverse | AACCTCCAATCCAGTCATCTAC |  |  |
| *VvGAPDH* ^b^ | Forward | TTCTCGTTGAGGGCTATTCCA | CB973647 | [57] |
|  | Reverse | CCACAGACTTCATCGGTGACA |  |  |

a = primers used for micropropagated periwinkle shoots; b = primers used for grapevine plants

**Supplementary File 5** Primers used in the experiments
